# Response Surface Methodology Analysis of Energy Harvesting System over Pathway Tiles

**DOI:** 10.3390/ma16031146

**Published:** 2023-01-29

**Authors:** P. Gothwal, A. Kumar, D. Rathore, R. Mukherji, T. Vetriselvi, S. Anandan

**Affiliations:** 1 Departments of Mechatronics Engineering, Manipal University Jaipur, Jaipur 303007, Rajasthan, India; 2 Department of IoT, School of Computer Science & E, VIT Vellore, Katpadi 632014, Tamil Nadu, India; 3 Amity School of Applied Sciences, Amity University Rajasthan, Jaipur 303007, Rajasthan, India; 4 Department of ECE, ICFAI University, Jaipur 302031, Rajasthan, India; 5 Department of Chemistry, National Institute of Technology, Trichy 620015, Tamil Nadu, India

**Keywords:** sensor, sustainable resource, PZT, electricity, energy harvesting, solar

## Abstract

This paper presents an experimental analysis of the optimization of PZT-based tiles for energy harvesting. The hardware (actual experiment), PZT-based tiles, were developed using 6 × 6 piezoelectric (PZT—lead zirconate titanate) sensors of 40 mm in diameter on a hard cardboard sheet (300 × 300 mm^2^). Our experimental analysis of the designed tiles obtained an optimized power of 3.626 mW (85 kg or 0.83 kN using 36 sensors) for one footstep and 0.9 mW for 30 footsteps at high tapping frequency. Theoretical analysis was conducted with software (Design-Expert) using the response surface methodology (RSM) for optimized PZT tiles, obtaining a power of 6784.155 mW at 150 kg or 1.47 kN weight using 34 sensors. This software helped to formulate the mathematical equation for the most suitable PZT tile model for power optimization. It used the quadratic model to provide adjusted and predicted R2 values of 0.9916 and 0.9650, respectively. The values were less than 0.2 apart, which indicates a high correlation between the actual and predicted values. The outcome of the various experiments can help with the selection of input factors for optimized power during pavement design.

## 1. Introduction

Global energy demand is surging due to dramatic urbanization, industrialization, and technological advancements [1]. The world’s energy consumption is expected to grow by 44% from 2006 to 2030 [2]. As India is the third-largest energy consumer across the globe, the energy challenges it faces are greater than in any other developing or developed country. India’s energy demand was expected to rise by almost 50% between 2019 and 2030, although since the COVID-19 pandemic, that prediction has been reduced to 35% over the same period [3]. A move toward cleaner energy production techniques including solar, wind, geothermal, etc., is conducive to meeting future energy challenges. Recently, energy harvesting from vibration energy using piezoelectric (PZT—lead zirconate titanate) floor tiles has been acknowledged by the technical community as one of the most promising new options. According to the principle of piezoelectricity, a potential difference is developed whenever the piezoelectric material experiences stress or mechanical pressure. Amongst the various mechanical stress sources, vibrations from human movements are used as the primary and spontaneous power source for PZT floor tiles, which convert them into useful energy [4].

Many researchers have studied PZT-based energy harvesting mechanisms. Ahad et al. [5] analyzed PZT sensors embedded in nine different materials and concluded that the PZT tiles with direct force produced greater voltages under foam and those with indirect force under aluminum. Abdal-Kadhim et al. generated energy via PZT transducers and achieved a maximum output voltage of approximately 50 V for 80 N force. Rumman et al. [6] developed tiles using PZT sensors for the mall to harvest the energy at the entrance gate by person movement. Taking a different approach, Kar et al. [7] presented a tire that could be used for energy harvesting and conducted a cost analysis and power estimation for a vehicle. The power production was observed at around 441 J, which was sufficient for low-power applications of the vehicle. Adhikari et al. [8] conducted a PZT performance analysis in terms of power using parameters such as frequency, tip mass, and EIDC. They noted that the output power increased 19-fold with an enhancement of EIDC values. Researchers striving to achieve energy neutrality also utilized a PZT-based energy generation concept [9,10,11,12]. Various reviews of novel PEH [13] for pavement applications generated an average output power of 3.106 mW, and importantly, noted that tiles should be protected by plastic material [14,15,16]. Various PEH for biomedical applications and nanogenerators with different material compositions have also been developed [17,18,19,20,21].

Sharpes et al. [22] also reported a novel approach for developing a new PZT material that can be utilized to function as a renewable resource. Table 1 presents a comparative analysis of various techniques for energy generation using PZT sensors.

The literature review revealed various ways to enhance the energy generated by PZT, such as with bending support, additional circuitry, a change to the number of sensors, and many more. By applying those, the present study aimed to design optimized PZT tiles with improved energy production. The present article details our experimental and theoretical analyses of the optimization of PZT-based tiles for energy harvesting through the response surface methodology (RSM).

Smart industries ensure that almost 90% of tasks are performed by robots to increase production. Hence, high consumption of electricity is required, and although solar panels are already installed in smart industries, both the current and future demand outstrip the provision of a single renewable energy source, meaning a mix of renewable resources must be incorporated. This paper presents the power optimization of PZT-based tiles, which can be assembled via traditional techniques and used at smart campuses, smart buildings, and smart offices, with the tiles placed in suitable locations such as ladders, lifts, corridors, etc.

## 2. Materials and Methods

Tiles were developed using PZT sensors of 40 mm diameter. The effect where piezoelectric material produces electricity on the application of a direct force is called the piezoelectric effect or piezoelectricity. Equation (1) represents the displacement under stress.

(1)  D→=dT+ϵTE

where D represents electric displacement vectors, T is stress, E is the electric field, *d* is the piezoelectric strain coefficient matrix, and ϵ is the dielectric permittivity. Two tiles were designed by placing PZT sensors over a platform. The platform for the tiles was made using a wooden or hard cardboard sheet to design model 1 (30 PZT sensors) and model 2 (36 PZT sensors), respectively, and this tile was laminated with plastic for protection. The experimental analysis of the tile involved testing it with three different cover materials: hard wooden board, steel plate, and cardboard sheet. Cardboard sheet was most suitable for generating optimized power. The material used to cover the tile was a laminated hard cardboard sheet of 2 mm thickness. The tensile strength was 10.8 kN/m and it had high stiffness.

### Experimental Details

**Figure 1:**
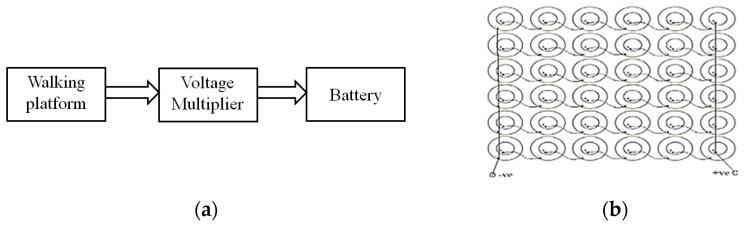
(**a**) Block diagram of PZT-based tiles’ energy generation, and (**b**) layout of walking tile platform.

A block diagram and the designed PZT tile for energy generation are shown in Figure 1a,b. In total, 36 piezoelectric sensors were installed on 300 × 300 mm^2^ hard cardboard sheet, as depicted in Figure 1b. The PZT sensors were arranged in a series-parallel configuration containing six rows and six columns, where every row contained six sensors, which were connected in series with each other, and these six rows were connected in parallel with each other. Furthermore, the tile was connected to a voltage multiplier circuit, which was designed using a capacitor and diode to stabilize the generated output voltage for battery storage.

## 3. Methodology

To create the affordable PZT tile, the required components are 36 PZT sensors, connecting wires, two hard cardboard sheets of 300 × 300 mm^2^ dimensions, a multimeter, and a glue gun. The hardness of the PZT sensor was 85, which was tested using a durometer under the applied force of a finger. The whole experimental setup was designed to generate optimized power. That included the arrangement of PZT disks in rows and columns, where the power generated (in voltage and current) under the applied load was tested using a multimeter and stored in a battery.

Two tiles were designed by placing PZT sensors over the platform. The platform for the tiles was made using wooden or hard cardboard sheet to design model 1 (30 PZT sensors) and model 2 (36 PZT sensors), respectively, where sensors were placed in series and with a parallel configuration. To make the arranged sensors immovable, the glue gun was used. A layer of flexible material rubber was utilized to cover the tops of the PZT disks. These designs were tested in different setups (one sensor, two sensors, four sensors, six sensors, 30 sensors, and 36 sensors), under varying loads, using different subjects, and by changing the connection of the sensors (series, parallel, and series-parallel). Pressure was applied on top of the solid cardboard sheet using varying force, from 4.6 kg to 85 kg, of the human body. The maximum power of 0.9 mW was recorded for one footstep with a maximum applied force of 85 kg. To begin with, two sensors, four sensors, and six sensors were tested only in series and with parallel connections separately. The corresponding maximum values of recorded power in terms of voltage and current observed. Next, the combination of series and parallel connections was tested using 30 and 36 PZT sensors and with weights from 29 kg to 85 kg.

### Statistical Assessment and Mathematical Modeling

Statistical techniques are employed to support and reinforce experimental research methodologies and findings. In the present work, RSM (response surface methodology) was applied to standardize the input variables (subject weight and number of sensors) to optimize the power. The designed tile was validated and optimized using statistical software, i.e., Design-Expert software version 13 (Stat-Ease, Inc., Minneapolis, MN, USA) and Microsoft Excel 2013. The recorded experimental data were analyzed and fitted to a quadratic equation with a regression coefficient. Through experimental analysis of the designed tile, we obtained an optimized power of 3.626 mW (85 kg or 0.83 kN using 36 sensors) for one footstep and 0.9 mW for 30 footsteps at high tapping frequency. Theoretical analysis was conducted with software (Design-Expert) using the response surface methodology (RSM) for PZT tile optimization, obtaining a power of 6784.155 mW at 150 kg or 1.47 kN weight using 34 sensors.

## 4. Results and Discussion

### 4.1. Software Validation and Optimization of Tiles

The response surface methodology (RSM) tool was used to design the theoretical experiments to optimize power. Two input parameters were used: sensor quantity (A) and subject weight (B). The RSM is a valuable statistical method that aids in the optimization of effective parameters with few experiments and the analysis of parameter interactions. The designed tile was validated and optimized using statistical software, i.e., Design-Expert software version 13 (Stat-Ease, Inc., Minneapolis, MN, USA). The analyses had two independent variables, i.e., sensor quantity (A) and weight (B), and one dependent variable, i.e., power (P).

**Figure 2:**
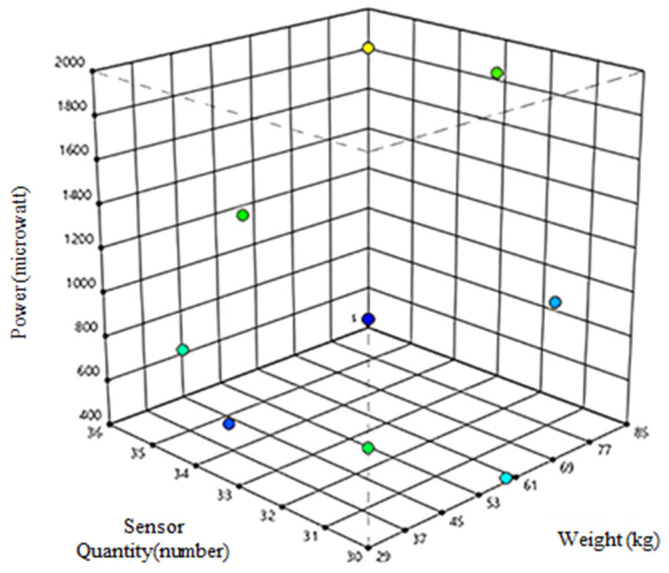
Correlation between power, sensor quantity, and weight.

According to the theoretical power analyses for A and B, the correlation factor was 0.494 or 0.740, respectively, as shown in Figure 2, which signifies that to optimize power, subject weight was more important than the number of sensors. The tool provides a theoretical design matrix with 13 experiments. These were conducted to study the effects of the two parameters (A and B) on power (P). The obtained experimental data were analyzed and fitted to a quadratic equation with a regression coefficient.

### 4.2. Development of Regression Model

**Figure 3:**
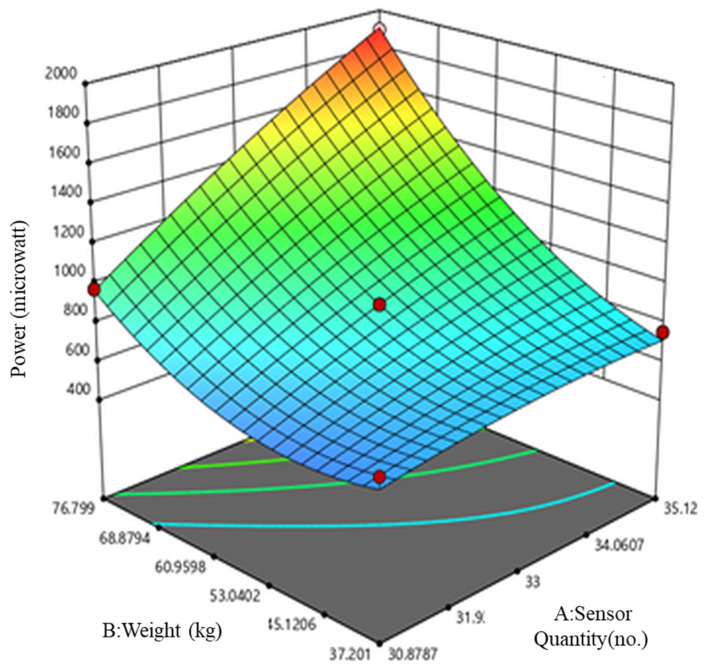
3D power response of the tile.

**Figure 4:**
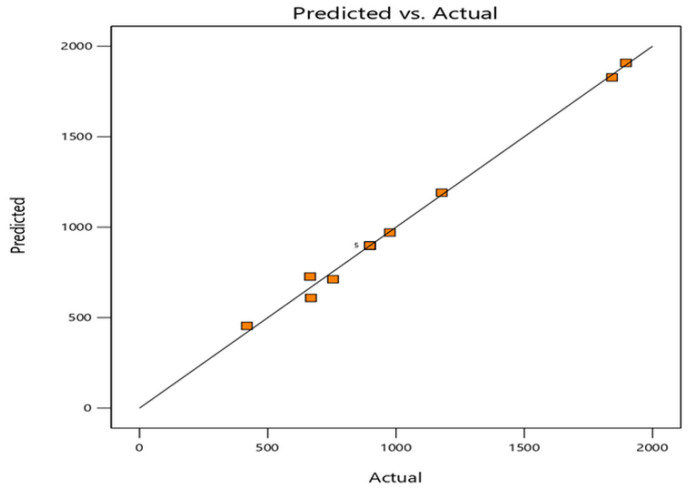
Power response graph of actual versus predicted model.

The 3D power response of the tile, measured by conducting experiments using operational parameters, is provided in the design matrix shown in Figure 3. The sequential model sum of squares shows the quadratic model to be best fitted to the theoretical data for power responses for A and B, and the cubic model is shown to be aliased for power responses. The Table 2 also reveals that the adjusted R2 value is 0.9916 and the predicted R2 value is 0.9650; their reasonable agreement of less than 0.2 indicates a high correlation between the observed and predicted values. In this theoretical analysis, there is only a single response optimized, i.e., power (P), as represented in quadratic Equation (2). A graph is also presented in Figure 4 showing the predicted and actual values of the experiments, which indicate the model’s suitability.

P = 898.31 + 260.14A + 389.32B + 208.58AB + 371.91A^2^ + 189.56B^2^ (2)

where A is the sensor quantity, B is the weight of the subject, and P is power.

### 4.3. Prototype Tile Validation

Two prototype models with different base materials and using 30 or 36 sensors were developed. The experiment with model 1 did not produce the desired power; therefore, model 2 was implemented using another base material (hard cardboard sheet with 36 sensors). Model 2 had different connections—series, parallel, and a series-parallel (P-S) configuration—which were tested under varying force (applied by different subjects). Initially, there were two, four, and six PZT sensors, connected in series and parallel, individually tested by applying different forces (29 to 85 kg) in different manners (via finger, single leg, and jumping). A maximum current of 16.7 µA in series and 62.9 µA for parallel connections were measured at 52 kg.

**Figure 5:**
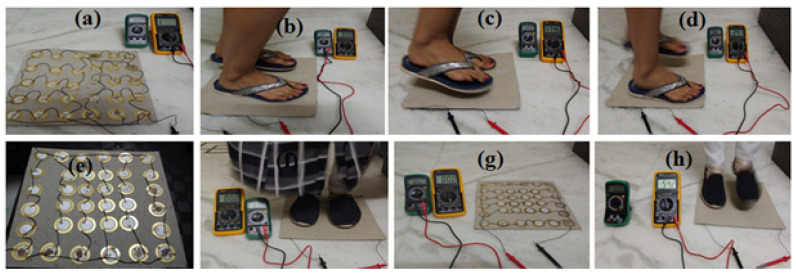
(**a**) Connection of 6 × 6 PZT sensors in series-parallel (S-P) configurations, (**b**) applied force of 65 kg with varying foot position 1, (**c**) applied force of 65 kg with jumping mode 1, (**d**) applied force of 65 kg with jumping mode 2, (**e**) connection of 6 × 6 PZT sensors in S-P configurations via changing alignment of sensors (CAS), (**f**) applied force of 65 kg with jumping mode position 1 via CAS, (**g**) connection of 6 × 6 PZT sensors in S-P configurations via CAS and connection and fixation of sensors via CAS, and (**h**) applied force of 65 kg with jumping mode position 2 via CAS.

**Figure 6:**
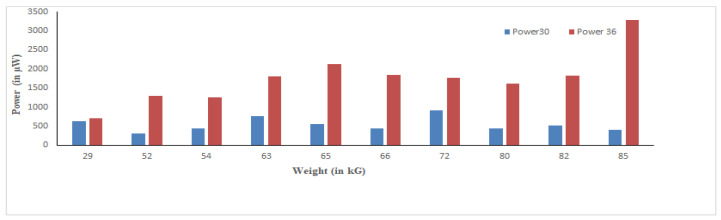
Actual power analysis using 30 and 36 sensors for different subject weights.

We observed that sensors connected only in parallel produced less voltage and more current, while sensors connected only in series produced more voltage and less current. Therefore, to optimize the maximum power, we designed a series-parallel combination of tiles. The results showed that by varying the load on designed tiles 1 and 2 (fast varying, stepping on platform, walking), the maximum power could be generated, as shown in Figure 5. The designed tiles were also tested for varying weights of different subjects, with the effects observed via the experimental setup illustrated in Figure 6. It can be concluded that weight is a key factor in generating maximum power. Hence, body weight and the generated output power of a designed tile have a direct relationship.

Furthermore, measurements were carried out by applying varying body force (using different people) and changing the alignment of the sensors (center and edges) with a P-S combination of 6 × 6 sensors, as shown in Figure 5. The maximum output power of 3.626 mW was achieved with 86 kg weight for one footstep using 6 × 6 sensors. In the next step, the generated power was passed through a voltage multiplier circuit to enhance the power stored in batteries.

### 4.4. Summary of Research Findings

**Figure 7:**
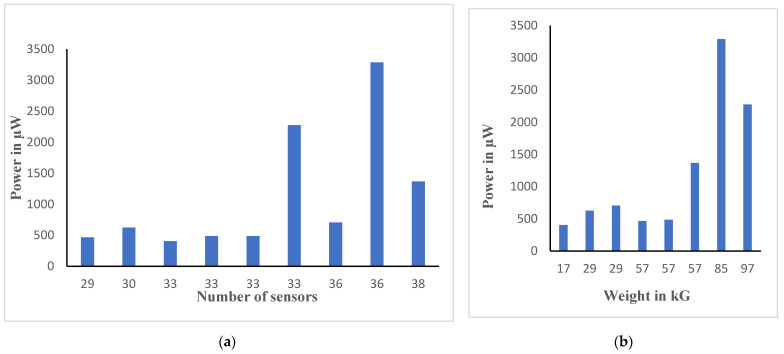
Predicted power analysis using varying quantities of (**a**) sensors and (**b**) weights of the subject.

The theoretical experiments using software as shown in Figure 7 revealed that optimized power (P) can be achieved by focusing on the subject weight (B), which has a higher correlation with power than the sensor quantity (A). Meanwhile, the practical experiments revealed that optimized power can be achieved by taking the following steps: (a) the PZT sensor should be securely fixed to a base material using a glue gun, (b) the tapping at varying force on the tile platform should be at a high frequency, (c) the PZT disk must be connected in an S-P configuration to achieve maximum power, (d) the PZT sensors must be in the center and at all four corners of the tile to generate the maximum voltage and current, (e) a large number of sensors and high body weight should be applied on the tile (series-parallel configuration), and (f) the designed tiles must be positioned in locations with high footfall, such as dancefloors and railway stations.

## 5. Conclusions

An affordable prototype model of a tile using 36 PZT sensors has been successfully designed and experimentally analyzed for maximum power generation with various combinations of sensors. The comparative experimental analysis tested two, four, and six PZT sensors individually connected in series and with a parallel configuration. We used a 6 × 6 matrix arrangement of sensors connected in a P-S configuration, where 6 sensors were connected in series and another 6 sensors were connected in parallel to generate the maximum power. The designed prototype tile (36 sensors) could generate a maximum power of 3.626 mW for an 86 kg load. In our experiments, we found the designed prototype tile could generate maximum power if it was placed in a realistic location, such as on a ladder, a disco floor, or a railway station. The maximum load limitation, tested with various subjects for the prototype tile, was 29–120 kg. The RSM analysis predicted a maximum load capacity of 150 kg for optimized power. Theoretical analysis was conducted with software (Design-Expert) using the response surface methodology (RSM) for PZT tile optimization. The obtained power was 6784.155 mW at 150 kg or 1.47 kN weight using 34 sensors. Hence, our experiments lead us to conclude that the tapping frequency, number of sensors, sensor fixation, sensor connection, and direction of applied force are important factors for maximizing output power. The theoretical experiments using RSM also revealed that the number of sensors (A) and subject weight (B) are crucial factors for optimizing power.

## Figures and Tables

**Table 1:** Comparative analysis of various available techniques for energy generation using PZT sensors.

Ref.	Technology	Number of Sensors	Output (Power, Voltage, and Current)	Force
[23]	PZT-based	-	50 V	80 N
[5]	PZT-based	72	15–18 V, 0.1 µA	-
[9,10]	PZT based	29, 30	6 µW	75 kG
[21]	PZT-based	54 with 9500 uF capacitor	Charges in 90 min up to 5.75 V at 4 Hz	-
[24]	PZT-based with additional circuitry	200	450 mW	
[25]	PZT-based	36	50 V	50 kG
[26]	PZT with bending mechanism	Single sensor with different diameters (30, 40, and 50 mm)	19, 34.4, and 50.4 V	3D printer technology
[27,28]	STEP technology using wireless-based PZT		Switch on the light and fan	Per footstep
	Novel PZT material		42 V and 11 μA	Per footstep
	Novel PZT with LED	10	300 μJ	-
[29]	S-sock using hybrid polymer and PZT chip	-	1.71 mW output power at 2 Hz and 59.7 MΩ	-
[30]	piezo sensor-based cantilever	-	energy (0.278 mJ)	one footstep
[31]	triboelectric energy harvester (TEH)		25 V for 0.5 g acceleration at 8 Hz	
[32]	PZT with boost converter	-	6.94 V for 300 mV input at 60 Hz AC	-
[33]	Piezoelectric (PE) and electromagnetic harvester (EM) for WSN		1–3.34 V	
[34]	44 PZTs with cantilever		35 mW_rms_	50 kG

**Table 2:** Power response fit summary for different models.

Source	Sequential *p*-Value	Adjusted R^2^	Predicted R^2^	Remarks
Linear	0.0004	0.7495	0.5596	
2FI	0.0448	0.8264	0.6868	
Quadratic	**<0.0001**	**0.96916**	**0.9650**	**Suggested**
Cubic	0.1133	0.9951	0.8683	**Aliased**
